# CRC-derived exosomes containing the RNA binding protein HuR promote lung cell proliferation by stabilizing c-Myc mRNA

**DOI:** 10.1080/15384047.2022.2034455

**Published:** 2022-02-07

**Authors:** Hui Xiao, Xiong Ye, Vikalp Vishwakarma, Ranjan Preet, Dan A. Dixon

**Affiliations:** a Department of Respiratory and Critical Care Medicine, Shanghai General Hospital, Shanghai Jiaotong University, Shanghai, China; b Department of Molecular Biosciences, University of Kansas Cancer Center, University of Kansas, Lawrence, Kansas, USA; c College of Clinical Medicine, Shanghai University of Medicine & Health Science, Shanghai, China

**Keywords:** colon cancer, human antigen R, exosomes, extracellular vesicle, proliferation

## Abstract

HuR overexpression is related to poor survival in patients with colon cancer. HuR overexpression leads to stabilization of tumor-promoting mRNAs by binding to 3′UTR-resident AREs. Exosomes, nanosized lipid bilayer vesicles, mediate many steps in cancer progression. The potential role of exosomal HuR in colon cancer lung metastasis is unclear. HuR expression was assessed immunohistochemically in tumor tissue samples from 20 patients with metastatic or nonmetastatic colon cancer and colon cancer lung metastasis and benign lung disease samples from ten patients. Exosomes were isolated from HCT116 WT and HuR KO colon cancer cells, and uptake of PKH67- and PKH26-labeled exosomes by BEAS-2B cells was evaluated using fluorescence and confocal microscopy. C-Myc and p21protein and mRNA levels were measured by western blotting and RT-qPCR, respectively. In clinical patients, HuR overexpression was significantly enhanced in colon tissues of patients with lung metastasis. HuR expression was higher in lung tissue with metastasis of colonic origin than with benign lung disease. The effect of HuR-containing CRC exosomes compared to HuR-deficient exosomes on wound closure was observed as enhanced proliferation. BEAS-2B cell migration and invasion were enhanced after HuR-containing exosomes treatment. BEAS-2B cells showed similar uptake of PKH67 (HCT116 WT)- and PKH26 (HCT116 HuR KO)-labeled exosomes. Exosomal HuR stabilized c-Myc mRNA and downregulated p21 expression, leading to G1/S transition, in human bronchial epithelial cells. HuR overexpression is associated with lung metastasis in colon cancer patients. Exosomal HuR derived from colon cancer cells alter the biological effect on normal lung epithelial cells.

## Background

Colon cancer is a systemic disease that causes more than one-quarter of all cancer-related deaths worldwide and metastasizes most frequently to the liver and lung.^1^ Distant metastasis is the major cause of death in patients with colorectal cancer (CRC).^2^ Thus, the identification of novel therapeutic targets to combat CRC metastasis is urgently needed to improve patient survival.

Exosomes are small (50–150 nm), membrane-bound, extracellular vesicles that are released by almost all types of cells, including stem cells,^3^ tumor cells,^4^ dendritic cells,^5^ mast cells,^6,7^ macrophages^8^ and natural killer cells.^9^ Exosomes can carry specific protein and RNA cargos that are present in the cell of origin and mediate diverse physiological and pathological functions in adjacent or remote cells by transferring their contents into the recipient cells; thus, exosomes mediate intercellular communication.^10–12^

The human antigen R (HuR) protein contains 3′ RNA binding domains and binds AU-rich elements. Because of these features, HuR is an important RNA binding protein that exerts pleiotropic effects on cell growth and tumorigenesis.^13,14^ Through its posttranscriptional influence on specific target mRNAs, HuR can alter the cellular response to proliferative,^15^ stress,^16^ apoptotic,^17^ differentiation,^18^ senescence,^19^ inflammatory^20^ and immune stimuli.^21^ HuR overexpression in colon cancer could constitute a regulatory pathway that controls mRNA stability and could contribute, via dysregulation of mRNA stability, to the progression of CRC.^22–24^ However, the role of HuR in colon cancer metastasis is unclear. The CDK inhibitor p21, also called p21waf1/cip1 (p21),^25^ is a well-known inhibitor of cell cycle progression and can induce G1/S arrest by inhibiting CDK2/cyclin E and cyclin A expression.^26^

Therefore, in this study, we hypothesized that HuR could influence lung cells via exosomes released from colon cancer cells. To test this hypothesis, we assessed the expression of HuR in colon cancer patients with distant metastasis and metastasis to the lung. In addition, we used a colon cancer cell line (HCT116) expressing HuR and a normal bronchial epithelial cell line (BEAS-2B) to determine whether exosomes can be taken up by bronchial epithelial cells and whether exosomal HuR can influence the function of these cells.

## Materials and methods

### Patients and clinical samples

The present study was approved by the Ethics Review Board of Shanghai First People’s Hospital Affiliated with Shanghai Jiaotong University (Shanghai, China). Written informed consent was obtained from all patients prior to enrollment in the study. All records were anonymized to protect individual confidentiality. Tumor tissues were retrieved from paraffin blocks. Clinical staging was performed according to the recommendations of the seventh edition of the American Joint Committee on Cancer staging system (AJCC-7) for colon cancer pathological staging.

### Immunohistochemical staining and immunoreactivity scoring

Resected specimens (colon cancer and lung tissue) were fixed in formalin and embedded in paraffin. Immunohistochemical analysis was carried out on 3-μm tissue sections. The sections were incubated with an anti-HuR/ELAVL1 rabbit monoclonal antibody (EPR17397, Abcam, China) at 4°C overnight. Then, the sections were incubated with a secondary antibody at room temperature for 60 minutes. The reagent from an ImmPRESS HRP Anti-rabbit Polymer Detection Kit (Vector Labs) was applied for 30 minutes. The reaction with horseradish peroxidase was visualized using ImmPACT DAB Peroxidase Substrate (Vector Labs). The sections were then counterstained with Mayer’s hematoxylin, dehydrated, cleared in xylene, and coverslipped using the mounting solution. Negative control of a colon cancer section processed without the primary antibody was established for every batch of slides to exclude nonspecific binding of the secondary antibody.

The immunoreactive staining of HuR in colon cancer tissue, lung metastasis tissue from colon and benign lung disease tissue was scored by applying a semi-quantitatively immunoreactive scoring (IRS) system. The staining intensity was scored as follows: 0 (negative staining), 1 (weak staining), 2 (moderate staining), or 3 (strong staining). The percentage of positive cells was scored as follows: 0 (no positive cells), 1 (< 10% positive cells), 2 (10–50% positive cells), 3 (51–80% positive cells), or 4 (> 80% positive cells). To calculate the immunoreactivity score (IRS), the two abovementioned scores were multiplied, resulting in values of 0, 1, 2, 3, 4, 6, 8, 9 or 12.

### Cell culture

The normal human bronchial epithelial cell line BEAS-2B was obtained from ATCC. BEAS-2B cells were maintained in LHC-9 medium (Gibco, USA) without FBS, and HCT116 cells were maintained in Dulbecco’s modified Eagle’s medium (DMEM; Corning, USA) supplemented with 10% exosome-depleted fetal bovine serum (Biological Industries), 100 U/ml penicillin and 100 μg/ml streptomycin. The flasks were kept in a humidified incubator at 37°C with 5.0% CO_2_. HCT116 cells were subjected to CRISPR/Cas9-mediated knockout of ELAVL1 gene. Stable HCT116 HuR knockout (KO) cells were grown in the same medium supplemented with puromycin (5 µg/ml).

### Exosomes isolation

The exosomes isolation procedure was described earlier.^22^ In brief, the culture medium from HCT116 cells was collected after 3 days of incubation. The medium was harvested and centrifuged first at 3,000 rpm for 10 minutes to remove cells and then at 10,000 rpm for 20 minutes (at 4°C) to remove cellular debris and larger vesicles. Exosomes were pelleted by ultracentrifugation at 120,000 × g for 70 minutes. The pellets were washed with ice-cold PBS and ultracentrifuged at 120,000 × g for 70 minutes. The exosomes pellets were resuspended in 150 μl of PBS and frozen at −80°C for further experimentation. The protein content of the exosomes was measured using the BCA protein assay kit (Thermo Scientific Pierce, Rockford, IL, USA).

### Transmission electron microscopy

In brief, a well-set grid was placed with the carbon film side down onto a 30 µl droplet of exosomes suspension and adsorbed for 20 minutes. After being washed with deionized distilled water, the grid was placed on a droplet of 4% uranyl acetate for 10 seconds. The grid was air dried for 5–10 minutes and was then observed under an electron microscope.

### Western blot analysis

Cells were lysed with lysis buffer. Then, the lysed proteins were separated by SDS-PAGE and transferred onto PVDF membranes (Immobilon-PSQ, Millipore, Billerica, MA, USA). The following antibodies were used: anti-CD81 (Santa Cruz Biotechnology, USA), anti-HSP90 (Abcam), anti-Calnexin (Abcam), anti-Flotillin-1 (Abcam), anti-HuR 3A2 (Santa Cruz Biotechnology, USA), anti-p53 (BD Transduction Laboratories, USA), anti-p21waf1/cip1 (Cell Signaling Technology Inc., USA), anti-Cdk2 (BD Transduction Laboratories, USA), anti-CyclinA (Cell Signaling Technology Inc., USA), anti-c-Myc (Cell Signaling Technology Inc., USA) and anti-β-Actin (Cell Signaling Technology Inc., USA). Antibody incubation was performed overnight at 4°C. Incubation with donkey anti-rabbit (IRDye® 680RD, LI-COR Biosciences, USA) and donkey anti-mouse (IRDye® 800CW, LI-COR Biosciences, USA) secondary antibodies was performed for 2 hours at room temperature. Blots were visualized using an Odyssey CLx Imaging System (LI-COR Biosciences, USA).

### Exosomes uptake assay

BEAS-2B cells were seeded (5 × 10^4^ cells/well) in chamber slides and incubated overnight. Fresh medium with 10 µg of dye-labeled exosomes (wild-type (WT) Exo-PKH67/KO Exo-PKH26) was added and incubated for 24 hours. Cells were rinsed with Hank’s balanced salt solution (HBSS)/0.01% sucrose and fixed with 2% paraformaldehyde (PFA). 4,6-Diamidino-2-phenylindole (DAPI; 10 µM) diluted in HBSS was added and incubated for 30 minutes. Cells were rinsed with HBSS and Vectashield mounting medium, and images were acquired by fluorescence microscopy. The cells were imaged using a spinning disk confocal microscope to confirm the presence of exosomes inside the cells. Exosomes uptake was quantified by determining the fluorescence intensity using ImageJ software.

### Wound healing assay

A total of 1 × 10^6^ cells/well were seeded in a six-well plate, and 90% confluence was ensured at the time of scratch wounding. The cells were pretreated with 0.5 μM mitomycin C to block cell proliferation for 2 hours. Mitomycin C is commonly used in cell migration assays to eliminate the confounding influence of cell proliferation. Three straight scratch wounds were made with a 10 µl pipette tip in each well, and nonadherent cells were removed by washing with culture medium. Fresh medium supplemented with TGF-β1(5 ng/ml) or WT/KO exosomes (WT/KO Exos20 μg/ml) were added to the wells, and the cells were incubated for up to 72 hours. The wound width was measured under a bright-field microscopic view immediately after wounding (0 hours) and at 24, 48, and 72 hours. The wound closure was measured by ImageJ software. All samples were tested in triplicate, and the data are expressed as the mean ± SEM values.

### Migration and invasion assays

Migration and invasion assays were carried out using 8.0 µm pore size Falcon cell culture inserts with or without a 10% Matrigel coating (BD Biosciences). In brief, 5 × 10^3^ BEAS-2B cells were seeded in the upper chamber, and fresh medium (500 µl) was added to the lower chamber for incubation overnight. The medium was changed to fresh medium, and later, the cells were stimulated with 5 ng/ml TGF-β1 (R&D Systems Minneapolis), incubated with medium alone, or stimulated with WT or KO exosomes (20 µg/ml) for 3 days. The cells remaining in the upper chamber were removed by wiping, and the remaining cells were gently washed with PBS. The adhered cells in the membrane were stained according to the Hema3 STAT Pack Kit (Thermo Fisher Scientific) instructions. Images were acquired, and the migrated and invaded cells were counted via crystal violet staining. At least five-microscope fields were counted for each condition.

### Cell viability assay

Cells were plated into a 96-well plate at 5,000 cells/well and incubated overnight. Cells were then treated with TGF-β1 (5 ng/ml), WT exosomes (20 µg/ml) or KO exosomes (20 µg/ml) for 24, 48 and 72 hours. MTT solution was added to the medium to a final concentration of 0.5 mg/ml and incubated for 4 hours at 37°C until intracellular purple formazan crystals were visible by microscopy. The MTT solution was removed, and DMSO was added and incubated at 37°C for 30 minutes until the cells had lysed and the purple crystals had dissolved. The absorbance was measured at 570 nm. Growth curves were drawn with doubling time calculated for each group by consecutive MTT assays for 72 hours. Each experimental group contained three replicate wells, and the experiment was repeated three times.

### Cell cycle analysis

BEAS-2B cells were seeded in 6-well plates overnight and were then stimulated with 5 ng/ml TGF-β1, incubated with medium alone, or stimulated with WT or KO exosomes (20 µg/ml) for 48 hours. Cells were harvested, and cellular DNA has stained with propidium iodide (PI) solution. Fluorescence was measured with a flow cytometer (BD FACSAria II, BD Bioscience, USA). Differences in cell cycle phases were distinguished on the basis of the DNA content. DNA histograms were analyzed with ModFit 5.0 software.

### RNA extraction and real-time reverse transcription-polymerase chain reaction (RT-PCR)

RNA was extracted using TRIzol reagent (Ambion Life Technologies, CA, USA), and first-strand cDNA was synthesized using SuperScript III reverse transcriptase (Invitrogen, Carlsbad, CA, USA). SYBER universal qPCR kits were used for real-time PCR. The PCR primers were as follows: p21 (forward 5′-GTG AGC GAT GGA ACT TCG A-3′, reverse 5′-AAT CTG TCA TGC TGG TCT GC-3′), c-Myc (forward 5′-TCA AGA GGC GAA CAC ACA AC-3′, reverse 5′-TAA CTA CCT TGG GGG CCT TT-3′) and GAPDH (forward 5′-CAA TGA CCC CTT CAT TGA CC-3′, reverse 5′-GAC AAG CTT CCC GTT CTC AG-3′). Three independent experiments were performed, and the data are shown as the mean ± SEM values.

### Statistical analysis

All values are expressed as the mean ± SEM of each experiment or independent cell culture-based experiments. All statistical analyses were performed using GraphPad Prism 6.0. The student’s t-test was used to analyze differences between the two groups. One-way ANOVA followed by Dunnett’s test was employed to compare differences among three or more groups. For all tests, significance was assumed at p ≤ .05.

## Results

### HuR was overexpressed in colon cancer patients with metastasis and in lung metastasis tissue of colonic origin

**Figure 1.**
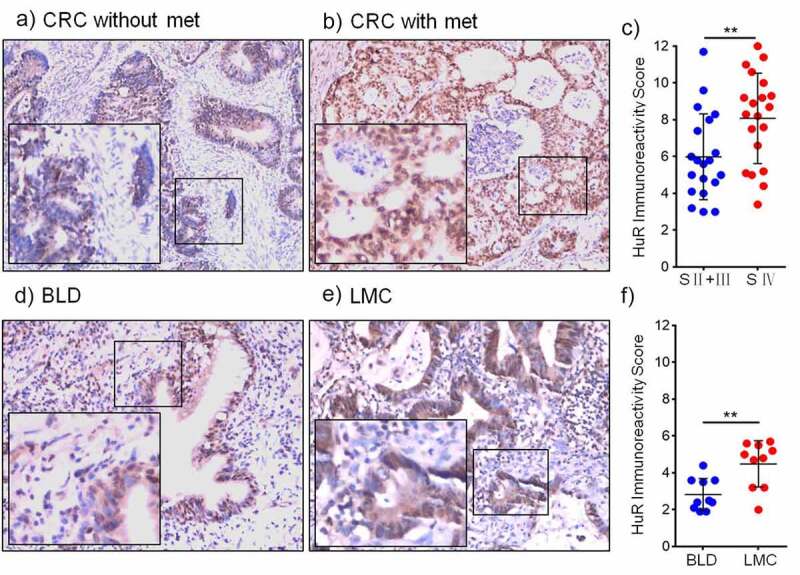
HuR expression in CRC patients with metastasis was detected using IHC. (a) CRC patients without metastasis (magnification, 200×) and (b) CRC patients with metastasis (magnification, 200×). (c) HuR IRS in CRC patients (stage II–III and stage IV). (d) HuR expression in benign lung disease and (e) HuR expression in lung metastases from the colon. (E) HuR IRS in lung metastases from the colon and benign lung disease. The student’s t-test was used to analyze differences between the two groups. The data are presented as the mean ± SEM value. **p < .01. BLD: benign lung disease, LMC: lung metastasis from the colon.

The immunohistochemical expression of HuR and the IRS are shown in Figure 1, which compares representative samples of colon cancer tissue from patients without metastasis (Figure 1a) and those with metastasis (Figure 1b). The samples of colon cancer with metastasis contained numerous HuR-positive cells. Therefore, overexpression of HuR was determined to be correlated with metastasis (p = .0085) (Figure 1c). Furthermore, immunohistochemical staining revealed that HuR was overexpressed in lung metastases from the colon compared to tissues of benign lung disease (Figure 1e). In samples of lung metastases from the colon, the numbers of HuR-positive cells were higher than those in the samples of benign lung disease (Figure 1d). figure 1f shows the IRSof HuR expression in patients with lung metastasis from the colon determined using immunohistochemistry (IHC) (p = .0029).

### Identification of exosomes derived from HCT116 WT and HuR KO cells

**Figure 2.**
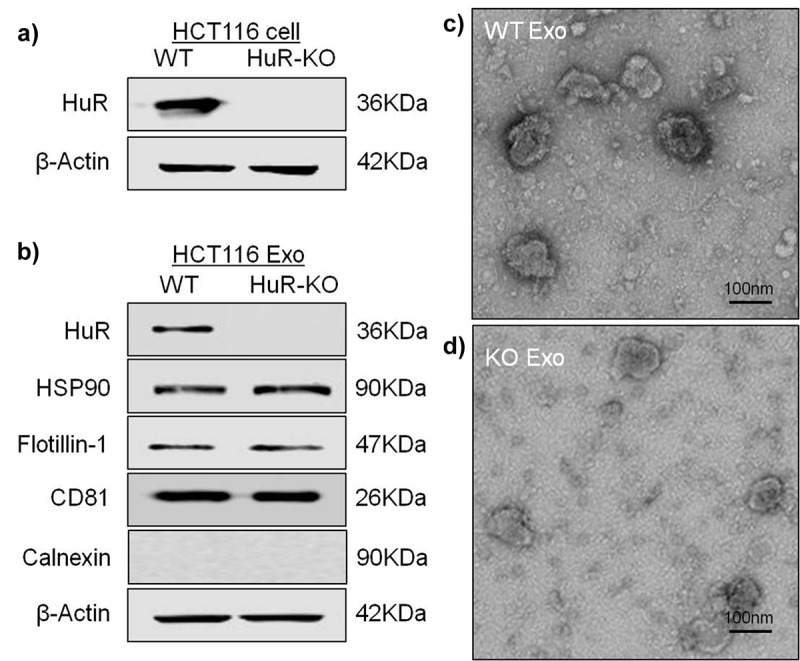
Identification and characterization of HCT116 exosomes. (a) Western blot analysis of HCT116 WT cells and HCT116 HuR KO cells showed the absence of the HuR protein in HCT116 HuR KO cells. (b) Western blot analysis of exosomes isolated from HCT116 WT and HuR KO cell supernatants showing the presence of the traditional exosomal markers CD81, HSP90 and flotillin-1, but absence of calnexin, in both cells lines, with absence of the HuR protein specifically in HuR KO cells. (c and d) Electron micrographs of the exosomes revealed rounded structures with a size of approximately 50–150 nm. The scale bar indicates 100 nm.

To confirm HuR KO in HCT116 cells, as shown in Figure 2a, we verified that HuR KO cells and exosomes did not contain HuR (Figure 2b). After isolation of exosomes from the supernatant of cultured HCT116 cells by ultracentrifugation, we observed cup-shaped structures between 30 and 150 nm by transmission electron microscopy (Figure 2c and d). The pellet after ultracentrifugation was positive for the traditional exosomal markers CD81, HSP90 and flotillin-1, but the absence of the endoplasmic reticulum protein, calnexin. (Figure 2b), verifying the identity of the exosomes^27^ and confirming our previous data showing that cancer cells can release exosomes.^12^ We also compared the protein and exosomes concentrations between WT and KO exosomes by the nanoparticle tracking analysis (NTA) and bicinchoninic acid (BCA) assay methods. As shown in this figure, there were fewer KO exosomes than WT exosomes (quantitative data not shown).

### Exosomal HuR enhances cell proliferation, migration, and invasion

**Figure 3.**
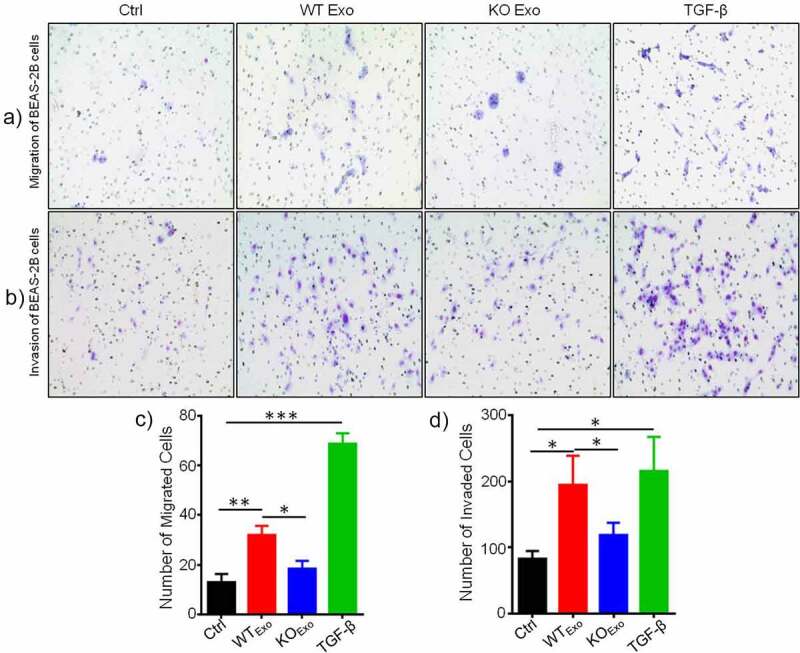
Exosomal HuR promotes the migration and invasion of BEAS-2B cells. Migration assay of BEAS-2B cells (5 × 10^3^ cells/well) incubated with HCT116 WT and HuR KO exosomes (20 µg/ml) or TGF-β1 (5 ng/ml) compared with LHC-9 medium as a control for 72 hours. Cells that migrated into the lower chamber (falcon cell culture insert) were photographed (a) and quantified (c). Matrigel invasion assay of BEAS-2B cells (5 × 10^3^ cells/well) incubated with HCT116 WT and HuR KO exosomes (20 µg/ml) or TGF-β1 (5 ng/ml) compared with LHC-9 medium as a control for 72 hours. Cells that invaded through the Matrigel were photographed (b) and quantified (d).TGF-β1 as a positive control. For each condition, cells were counted from 5 different fields. The student’s t-test was used to analyze differences between the two groups. *p < .05, **p < .01, ***p < .001. The data are presented as the mean ± SEM values. All experiments were repeated at least three times.

**Figure 4.**
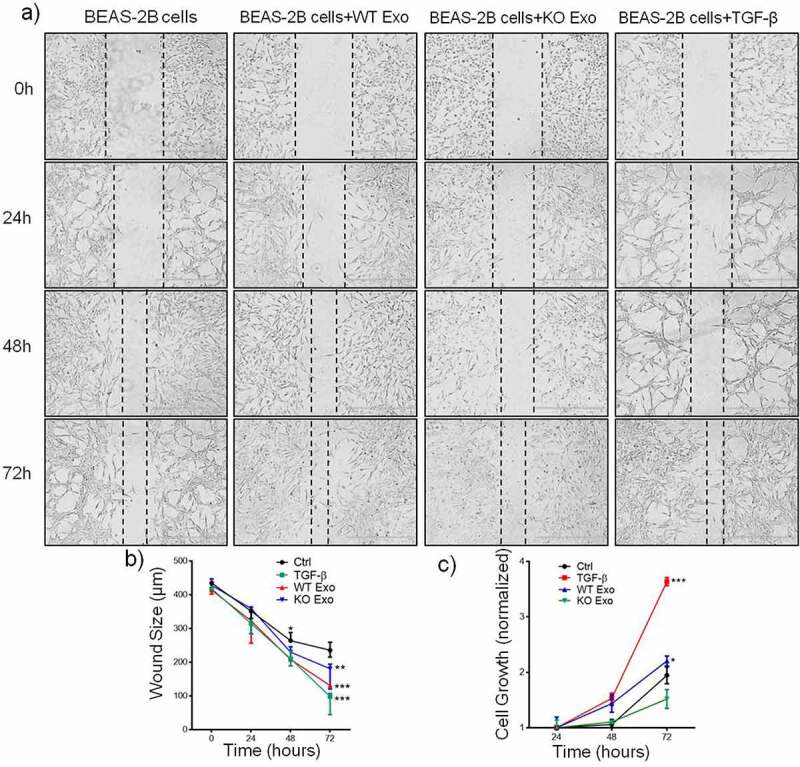
Exosomal HuR promotes the migration and proliferation of BEAS-2B cells. Alterations in migration induced by HCT116 WT and HuR KO exosomes (20 µg/ml) or TGF-β1 (5 ng/ml) were evaluated by a wound healing assay (a and b). (c) An MTT assay was performed to evaluate the proliferation of BEAS-2B cells stimulated with HCT116 WT or HuR KO exosomes (20 µg/ml) or TGF-β1 (5 ng/ml). Scale bar, 400 µm. The student’s t-test was used to analyze differences between the two groups. One-way ANOVA followed by Dunnett’s test was used to analyze differences among three groups. *p < .05, **p < .01, ***p < .001. The data are presented as the mean ± SEM values. All experiments were repeated at least three times.

Previous data showed that exosome-mediated transport of functional proteins into cancer cells can lead to proliferation and metastatic properties in lung cancer.^7^ Therefore, we then explored the role of exosomal HuR and found that it enhanced the migration and invasion of BEAS-2B cells compared with untreated cells and cells treated with KO exosomes (Figure 3a and b). TGF-β was added to the cultured cells as a positive control. Statistical results are shown in Figure 3c and d. Next, we evaluated the effect of exosomal HuR on the migration, chemotaxis and colony formation capacities of BEAS-2B cells. Wound healing assays showed that the migratory activity of BEAS-2B cells treated with WT exosomes was significantly higher than that of control cells (Figure 4a and b). Similarly, HCT116 WT exosomes carrying HuR promoted the proliferation of BEAS-2B cells (Figure 4c).

### Uptake of exosomes derived from HCT116 cells by BEAS-2B cells

**Figure 5.**
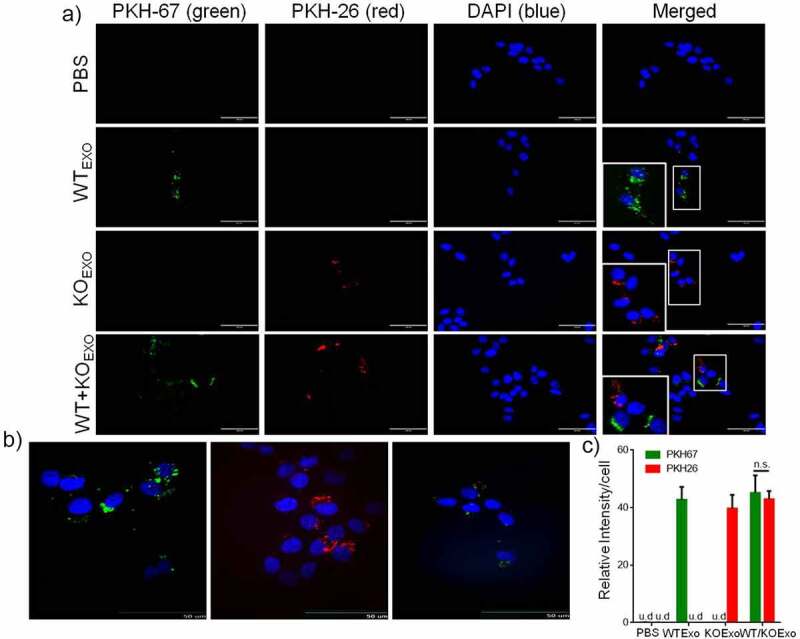
Uptake of HCT116 WT and KO exosomes by BEAS-2B cells. Ten micrograms of PKH67-labeled HCT116 WT exosomes, PKH26-labeled HCT116 HuR KO exosomes or a PKH67/26-PBS control were added per 5 × 10^4^ BEAS-2B cells and incubated at 37°C for 24 hours. Uptake of the fluorescently labeled exosomes by BEAS-2B cells was detected with fluorescence microscopy and confocal microscopy (at 24 hours). (a) Uptake of PKH67/26-PBS control, PKH67-labeled HCT116 WT exosomes (green) and PKH26-labeled HCT116 HuR KO exosomes (red) in BEAS-2B cells at 37°C was evaluated with fluorescence microscopy. Nuclei were stained with DAPI (blue) (scale bar, 100 μm). (b) Uptake of PKH67-labeled HCT116 WT exosomes and PKH26-labeled HCT116 HuR KO exosomes was evaluated with spinning disc confocal microscopy (scale bar, 50 μm). Nuclei were stained with DAPI (blue). (c) Exosomes uptake was quantified by determining the fluorescence intensity, and intensity values are shown as the means ± SEMs (N = 3). The student’s t-test was used to analyze differences between two groups.n.s., no significant difference; u.d., undetected.

To evaluate whether exosomes from HCT116 cells can be taken up by BEAS-2B cells, exosomes from HCT116 cells and HCT116 HuR KO cells were labeled with PKH67 dye and PKH26 dye, respectively, and were then added to cell cultures. PBS containing PKH67 and PKH26 dye was used as the control treatment. Figure 5a shows fluorescence images after exosomes uptake by BEAS-2B cells for 24 hours. Exosomes uptake was quantified by determining the fluorescence intensity. Exosomes internalization was not dependent on the presence of HuR in the exosomes, and the exosomes localized in the cytoplasm of BEAS-2B cells, as shown by the confocal images (Figure 5b). Figure 5c shows that there was no difference in the uptake of WT and KO exosomes by BEAS-2B cells.

### HuR regulates p21 mRNA transcription and protein expression to promote cell cycle progression

**Figure 6.**
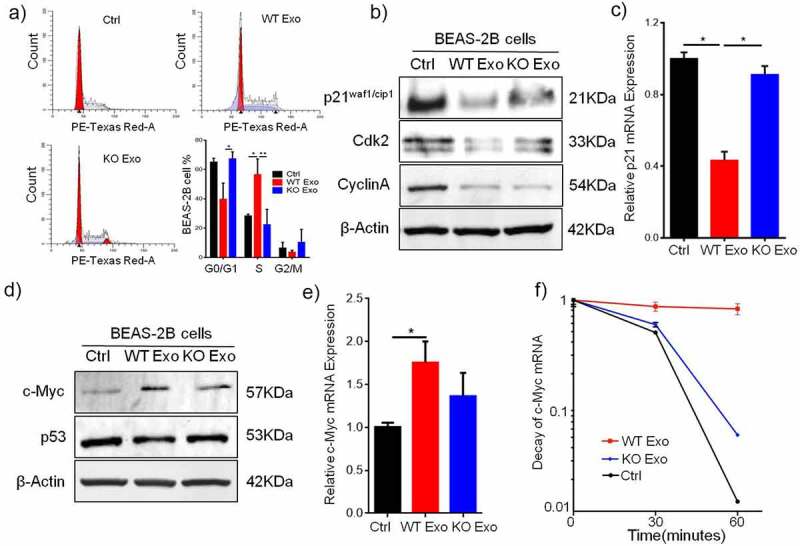
Exosomal HuR promotes cell cycle progression in BEAS-2B cells by inhibiting p21 expression. (a and b) Cell cycle histograms of unexposed BEAS-2B cells and BEAS-2B cells incubated with HCT116 WT exosomes or HuR KO exosomes (20 µg/ml). (c) BEAS-2B cells were incubated with HCT116 WT or HuR KO exosomes (20 µg/ml). p21, Cyclin A and Cdk2 expression was assessed by Western blotting, and to normalize protein loading, samples were also probed for β-Actin. (d) Relative p21 mRNA expression in BEAS-2B cells treated with HCT116 WT or HuR KO exosomes (20 µg/ml) for 72 hours. (e) c-Myc and p53 expression were assessed by Western blotting, and to normalize protein loading, samples were also probed for β-Actin. (f) Relative c-Myc mRNA expression in BEAS-2B cells treated with HCT116 WT or HuR KO exosomes (20 µg/ml) for 72 hours. (g) The decay rates of c-Myc mRNA and GAPDH in BEAS-2B cells, as indicated, were assessed by RT-qPCR after treatment with HCT116 WT or KO exosomes for 72 hours and inhibition of transcription with actinomycin D. The data are presented as the mean ± SEM values. The student’s t-test was used to analyze differences between the two groups.*p < .05, **p < .01; n = 3.

P21, a cyclin-dependent kinase inhibitor, plays a key role in regulating the cell cycle by interacting with cyclin–CDK complexes and directly inhibiting their kinase activity. To assess the regulatory effect of exosomal HuR on the cell cycle, we assumed that changes in p21 expression might be a possible mechanism leading to G1/S transition during growth induced by exosomal HuR. Consistent with this hypothesis, flow cytometric analysis showed that exosomal HuR (20 µg/ml) increased the number of cells in S phase compared to that among untreated cells or cells treated with HuR KO exosomes (Figure 6a). Next, we examined whether the decrease in p21 protein expression mediated by exosomal HuR is correlated with a decrease in p21 transcription (Figure 6b). To this end, RT-qPCR was performed to assess p21 expression in BEAS-2B cells that were untreated or treated with exosomes for 2 days (Figure 6c). Furthermore, p21 decreased the expression of Cdk2 and cyclin A, as shown by Western blot analysis (Figure 6b). In treated cells, exosomal HuR treatment led to a 2-fold decrease in the p21 mRNA level (Figure 6c).

### HuR stabilizes c-Myc mRNA expression to inhibit p21 expression

Interestingly, p21 expression was independent of p53 expression during the cell cycle and growth, and c-Myc expression was upregulated by HuR carried in WT exosomes to inhibit the expression of p21 mRNA and protein (Figure 6d and b). In addition, c-Myc mRNA expression in BEAS-2B cells was increased by HuR-containing exosomes compared with HuR KO exosomes (Figure 6e). Next, to examine the c-Myc mRNA half-life (*t*_1/2_) in BEAS-2B cells treated with WT/KO exosomes after treatment with the transcription inhibitor actinomycin D, the quantity of c-Myc mRNA was measured by RT-qPCR at 0, 30, 60 and 120 minutes. In WT exosome-treated BEAS-2B cells, the c-Myc mRNA *t*_1/2_ was 96 minutes, whereas in KO exosome-treated BEAS-2B cells, the c-Myc mRNA *t*_1/2_ decreased to 33 minutes. The *t*_1/2_ of GAPDH mRNA, as the control, was not changed after treatment with WT exosomes (figure 6f). Therefore, these results indicate that c-Myc mRNA was stabilized by HuR, which inhibited p21 expression in BEAS-2B cells.

**Figure 7.**
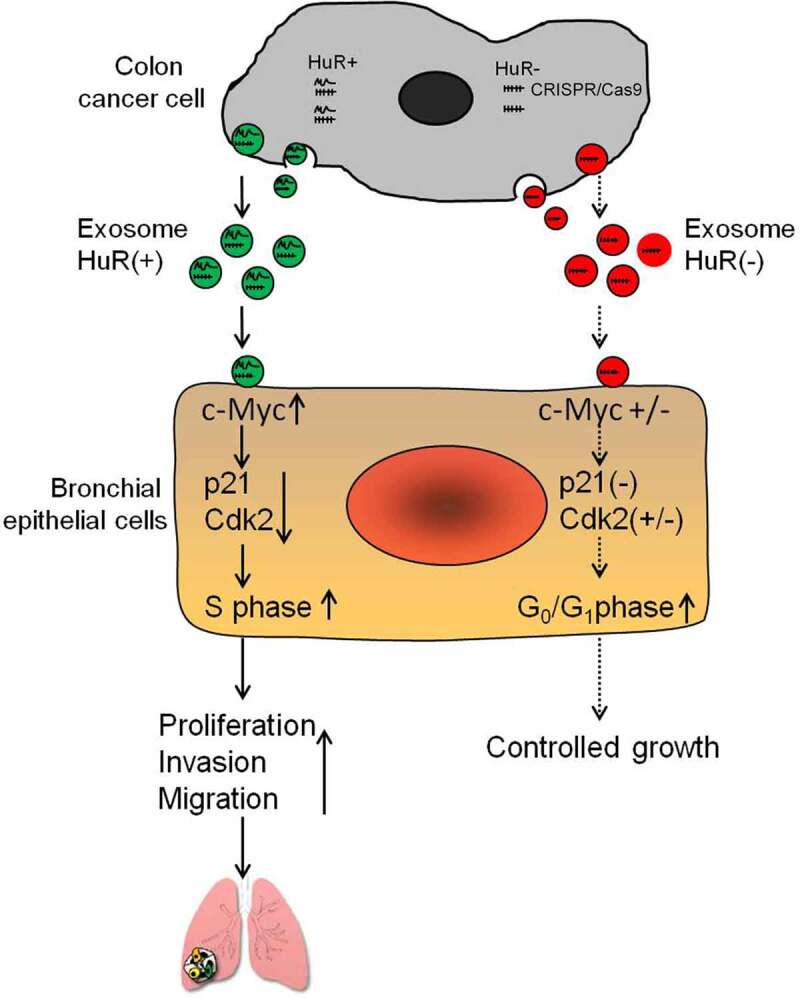
Schematic diagram showing that colon cancer cells can release exosomes that can be taken up by BEAS-2B cells. In BEAS-2B cells, these exosomes can stabilize c-Myc mRNA and influence the expression of p21. p21 expression, therefore, decreases in the cells and accelerates cell cycle progression. Eventually, exosomal HuR from colon cancer cells causes proliferation, migration and invasion of lung cells.

As a summary, Figure 7 presents a schematic diagram showing that colon cancer cells can release exosomes containing HuR, which is taken up by bronchial epithelial cells. In turn, exosomal HuR affects the activity and function of the cell cycle-dependent kinase inhibitor p21 in bronchial epithelial cells, which leads to the invasion and migration of these cells. Therefore, HuR carried in exosomes derived from colon cancer cells ultimately promotes proliferation, migration and invasion of normal lung cells.

### Discussion

Metastasis is the leading cause of death in patients with colon cancer, and little is known about how metastasis clinically influences cancer development. Research suggests that exosomes can transfer RNA, protein and other molecules between cells, a process likely to be very active in intercellular signaling in tumors. HuR is the key molecule in cancer progression. However, little is known about the mechanisms by which HuR might specifically promote cancer metastasis. In this study, expression of HuR was significantly associated with metastasis, particularly lung metastasis from the colon, in clinical patients. Moreover, we found that exosomes derived from the human colon cancer cell line HCT116 carrying the HuR protein can be taken up by bronchial epithelial cells (BEAS-2B). Furthermore, the uptake process was independent of the presence of the HuR protein. Our results suggest that exosomal HuR can suppress p21 protein expression and mRNA transcription in BEAS-2B cells, which in turn promotes cell cycle progression, proliferation, migration, and invasion. Collectively, these data suggest that HuR is overexpressed in colon cancer patients and contributes to the potential mechanism underlying lung metastasis of CRC via exosomes.

In this study, we confirmed that HuR is overexpressed in colon cancer patients with distant metastasis, including lung and liver metastasis, compared with CRC patients without metastasis. In addition, the HuR IRS in lung metastases from the colon differed markedly from that in the tissue of benign lung diseases, such as benign lung nodules and chronic obstructive pulmonary disease. These findings emphasize that colon cancer is a complex disease; in particular, lung metastasis from the colon is a very important step in its progression. These findings suggest that HuR is overexpressed in patients with colon cancer with the highest risk of metastasis and that HuR expression is also high in patients with lung metastasis from the colon.

In the present study, we identified the ability of the colon cancer cell line HCT116 to release exosomes and extended this finding by demonstrating the ability of these exosomes to be taken up by human bronchial epithelial cells (BEAS-2B). A key finding of the present study was that uptake by BEAS-2B cells was not dependent on the presence of HuR. In other words, the amount of exosomes taken up by BEAS-2B cells was the same for both HCT116 WT exosomes and HCT116 HuR KO exosomes. Furthermore, HuR was confirmed to be localized in the cytoplasm of BEAS-2B cells by confocal microscopy.

Less is known about the functionality of exosomal HuR than endogenous HuR. The current experiments showed the wound healing, proliferation, migration and invasion abilities of BEAS-2B cells after the addition of exosomes. We confirmed that the addition of HuR-containing colon cancer cell-derived exosomes enhanced the proliferation of BEAS-2B cells and promoted their migration and invasion. In contrast, exosomes derived from cells with HuR knockout did not dramatically enhance proliferation, migration, or invasion. In addition, we found that HuR KO exosomes also changed these functions slightly compared with those in the control cells. There was more TGF-β1 in the exosomes derived from the HCT116 HuR KO cells than in the HCT116 WT exosomes, but the difference between the groups was not significant. However, this hypothesis related to TGF-β1 is outside the scope of the present study.

Here, we evaluated the cell cycle in BEAS-2B cells incubated with exosomes and explored the potential mechanism. The addition of exosomes carrying HuR derived from colon cancer cells to BEAS-2B cells accelerated cell cycle progression through the G1/S transition; comparatively, cells treated with HuR-negative exosomes or the control exhibited G1 arrest. The Cdk inhibitor p21 is a well-known regulator of the cell cycle and can arrest the cell cycle at the G1/S and G2/M transitions by inhibiting Cdk2 and cyclins.^28–30^ In this study, we showed that p21 was inhibited and Cdk2 was downregulated. Collectively, these data suggest that HuR-containing colon cancer cell-derived exosomes can indeed inhibit signaling cascades downstream of p21. We therefore propose that colon cancer cell-derived exosomes carrying HuR may inhibit p21 transcription in recipient bronchial epithelial cells and thus inhibit the protein expression of p21. This can, in turn, allow the molecule to function as a cell cycling promoter by driving cell cycle progression from G1 phase to S phase. Collectively, our data imply that HuR-containing exosomes derived from colon cancer cells can influence the p21 signaling pathway in bronchial epithelial cells.

HuR was found to strongly associate with the 3′-UTR of c-Myc mRNA and block mRNA translation. The binding efficiency of HuR to the 3′-UTR of c-Myc mRNA was confirmed by Western blot analysis, RT-PCR and decay assays. Kim et al proposed that HuR recruits let-7/RISC to repress c-Myc expression;^31^ therefore, this hypothesis requires further investigation. A study by Hyeon et al showed that HuR and let-7 repress c-Myc through an interdependent mechanism, as HuR required let-7 to inhibit c-Myc expression. However, Talwar et al revealed that HuR underwent posttranslational modifications and recruited c-Myc mRNA during hypoxic stress.^32^ This observation further corroborated the findings of our study.

Indeed, this study has limitations. In our clinical specimen analysis, the patients with stage IV colon cancer included a subset of patients with liver metastases and lung metastases. In the in vitro experiments, the detailed mechanism of HuR KO exosomes was not further explored.

In summary, our data showed that HuR overexpression in colon cancer patients is related to a higher risk of metastasis, particularly to the lung. Furthermore, our study showed that HuR carried by exosomes derived from colon cancer cells can enhance the proliferation, migration, and invasion of bronchial epithelial cells (BEAS-2B), which inhibited the expression of p21 in these cells. Based on our results, further studies are necessary to explore whether HuR might be a molecular therapeutic and diagnostic target as well as a prognostic marker for colon cancer.

## Figures and Tables

**Table:** 

FBS	fetal bovine serum
HuR	human antigen R
IRS	immunoreactivity scoring system
CRC	colorectal cancer
GAPDH	glyceraldehyde 3-phosphate dehydrogenase
CDK	cyclin-dependent kinase
3′ UTR	3′ untranslated region
